# Embracing the Future of Medical Education With Large Language Model–Based Virtual Patients: Scoping Review

**DOI:** 10.2196/79091

**Published:** 2025-11-13

**Authors:** Jianwen Zeng, Wenhao Qi, Shiying Shen, Xin Liu, Sixie Li, Bing Wang, Chaoqun Dong, Xiaohong Zhu, Yankai Shi, Xiajing Lou, Bingsheng Wang, Jiani Yao, Guowei Jiang, Qiong Zhang, Shihua Cao

**Affiliations:** 1 School of Public Health and Nursing Hangzhou Normal University Hangzhou, null China; 2 Zhejiang Provincial Research and Evaluation Center for Educational Modernization Hangzhou, null China; 3 Department of Psychiatry and Neuropsychology and Alzheimer Center Limburg, School for Mental Health and Neuroscience (MHeNS) Maastricht University Maastricht The Netherlands; 4 Department of Nursing Zhejiang Provincial People's Hospital Hangzhou China

**Keywords:** artificial intelligence, virtual patient, large language model, medical education, scoping review

## Abstract

**Background:**

In recent years, large language models (LLMs) have experienced rapid development. LLM-based virtual patients have begun to gain attention, offering new opportunities for simulations in medical education.

**Objective:**

This study aims to systematically analyze the current applications, research trends, and challenges of LLM-based virtual patients in medical education and to explore potential future directions for development.

**Methods:**

This study adheres to the PRISMA-ScR (Preferred Reporting Items for Systematic Reviews and Meta-Analyses extension for Scoping Reviews) guidelines. Five databases (Web of Science Core Collection, PubMed, IEEE Xplore, Embase, and Scopus) were searched from January 1, 2018, to June 24, 2025, to identify studies related to the application of LLM-based virtual patients in medical education. A comprehensive analysis of LLM-based virtual patients from research design to application and evaluation was conducted.

**Results:**

A total of 28 studies were included in this scoping review. Analysis revealed that 92.9% (26/28) of the studies were published in the past 2 years, indicating that LLM-based virtual patient research is still in its early stages. The research primarily focuses on medical training and spans a wide range of medical disciplines. When using LLMs, advanced technologies such as social robots, virtual reality, and mixed reality are used to present LLM-based virtual patients. Combining these technologies with various supplementary tools enhances the realism of LLM-based virtual patients and improves user interaction. The evaluation of LLM-based virtual patients mainly emphasizes user experience. However, evaluation methods lack standardization, and only 13% (3/23) of studies used validated tools in assessing LLM-based virtual patients, while only 21.7% (5/23) of studies objectively measured learning outcomes facilitated by LLM-based virtual patients. All included studies expressed a positive attitude toward LLM-based virtual patients; however, they overlook privacy and security considerations in practical applications.

**Conclusions:**

LLM-based virtual patients hold significant innovation potential in medical education and are still in the early stages of development. They are primarily applied in medical training and show promise in communication skills training, although they cannot replace real-world interactions. Moreover, the heterogeneity of research designs, the absence of nonverbal cues in interactions, and concerns regarding privacy and security limit their broader implementation. Future research should focus on improving the reliability, realism, safety, and scientific efficacy of LLM-based virtual patients.

**Trial Registration:**

Open Science Framework Registries 10.17605/OSF.IO/DMC9Q; https://osf.io/DMC9Q/overview

## Introduction

In recent years, large language models (LLMs) have made significant progress [1,2]. LLMs are high-performance artificial intelligence (AI) systems capable of understanding and generating natural language [3]. With advancements in AI, LLMs have demonstrated great potential in tasks involving natural language processing [4]. Their applications range from text analysis and summarization to clinical applications, showcasing their flexibility in providing valuable assistance [5-7]. LLMs support user interactions through follow-up questions and are fine-tuned to generate controlled outputs [8,9]. More importantly, they allow developers to create chatbots and virtual assistants with customized behaviors [9]. Given these capabilities, LLMs are expected to become efficient and feasible tools across various domains, including medical education, where traditional virtual patients have also been used.

Virtual patients are computer-based programs that simulate real clinical scenarios, allowing learners to take on the role of health care professionals. The goal is to develop skills and knowledge in specific areas while enabling learners to practice decision-making in a controlled interactive environment [10,11]. Virtual patients have been shown to be effective in teaching, assessment, and clinical reasoning research [12] and have been proposed as a valuable educational tool for practicing clinical reasoning in undergraduate medical education [13]. They play a crucial role in medical education.

However, the application of virtual patients faces logistical challenges and high costs for large-scale implementation [14]. For instance, studies have shown that the technological development cost of a virtual patient is US $12 per hour, with the average monthly cost of developing and maintaining a virtual patient system being US $324.75 [15,16]. These limitations often prevent all students from engaging in interactive skill practice or performing multiple exercises, significantly reducing the effectiveness of the practice. However, the emergence of LLMs as a disruptive technology offers unprecedented opportunities to overcome the limitations faced by traditional virtual patients. LLM-based virtual patients combine natural language processing technology with medical knowledge, using LLMs to construct virtual avatars. These avatars are designed to simulate the behavior, symptoms, diagnostic processes, and disease progression of real patients, creating highly realistic and diverse virtual patient models [17-19]. They can present standardized patients in various scenarios, supporting students’ clinical reasoning, decision-making, and problem-solving skills while also providing performance analysis and feedback [20,21]. Although LLMs face inherent limitations and negative impacts in practical applications, such as “hallucinations” [22] and influences on independent thinking [23], these challenges do not prevent LLMs from offering new opportunities in medical education simulations.

Recent research has discussed the application of traditional virtual patients, with some systematic reviews highlighting the positive impact of virtual patient simulators on medical communication training. These reviews emphasize the adaptability of virtual patients and their value as a supplement to traditional educational methods [24,25]. However, to date, no study has comprehensively summarized the application of LLM-based virtual patients in medical education. This paper aims to provide a comprehensive overview of the positioning, challenges, and future directions of LLM-based virtual patients in medical education, offering a reference for the better development and application of LLM-based virtual patients.

As an innovative and transformative technology, LLM-based virtual patients demonstrate tremendous potential in medical practice and are expected to drive the field toward greater efficiency, precision, and personalization. To comprehensively analyze their current applications, technological challenges, and future directions, this paper focuses on the following key issues: (1) In which areas of medical education are LLM-based virtual patients primarily applied? Which medical disciplines are involved, and what are the main research directions? (2) What are the primary LLMs currently used? How are the models fine-tuned, and what is the role of prompt engineering? (3) How are LLM-based virtual patients specifically implemented in practical applications? Specifically, how is the instructional design (application scenarios and learning activity design), technological design (integrated technology ecosystem, interaction modes, and auxiliary tools), and assessment design (user experience, learning outcomes assessment, evaluation standards, and evaluation roles) structured? (4) What are the key challenges faced by LLM-based virtual patients, and what are the future research directions?

## Methods

### Study Design

This study uses a scoping review methodology due to the diversity of the research questions, the heterogeneity of the studies, and the lack of comprehensive previous reviews on this topic [26,27]. The scoping review framework follows the approach proposed by Arksey and O’Malley [26] and is reported according to the PRISMA-ScR (Preferred Reporting Items for Systematic Reviews and Meta-Analyses extension for Scoping Reviews) checklist for scoping reviews [27]. The complete PRISMA-ScR checklist is available in Multimedia Appendix 1. This scoping review has been registered in the Open Science Framework (10.17605/OSF.IO/DMC9Q).

Several factors led to deviations from the original protocol regarding inclusion and exclusion criteria. First, given that AI is a rapidly evolving field and high-impact journals frequently publish important peer-reviewed original research in the form of “research letters,” we decided not to exclude conference papers and letters. Second, considering the growing attention on compact LLMs with fewer than 10 billion parameters, we removed the restriction on the model size in the inclusion criteria. Additionally, due to potential difficulties in retrieving non-English literature, and given that English-language publications adequately cover key developments in the fields of natural sciences and medicine, we decided to exclude non-English papers.

### Data Sources and Search Strategy

To ensure comprehensive retrieval and consider the interdisciplinary nature of LLM-based virtual patients in medical applications, a literature search was conducted across 5 major databases: PubMed, Web of Science Core Collection, Scopus, Embase, and IEEE Xplore. We collaborated with librarians and medical informatics experts to develop the search strategy. To ensure thoroughness and minimize the risk of missing relevant literature, the core search terms included 2 categories: one related to “generative AI” and “large language models” and the other related to “virtual patients,” using Boolean operators (eg, AND and OR) for combination. Additionally, we reviewed the reference lists and citations of relevant papers. The search time frame was from June 2018 to April 24, 2025. June 2018 marks the release of the first generative AI model [28]. Literature management and duplicate removal were conducted using EndNote (version 20; Clarivate Analytics) software. A detailed description of the search strategy can be found in Table S1 in Multimedia Appendix 2.

### Inclusion and Exclusion Criteria

The inclusion and exclusion criteria are listed in Textbox 1.

**The inclusion and exclusion criteria.**

**Inclusion criteria**

- The literature must focus on research related to large language model–based virtual patients.
- The literature must explicitly address the application of large language model–based virtual patients in medical education, including technical development or practical case studies.
- Eligible types of literature include journal papers, conference papers, and research letters.

**Exclusion criteria**

- Studies for which the full text is not accessible.
- Duplicate publications.
- Conference abstracts, preprints, books, editorials, reviews, and retracted studies.
- Studies unrelated to medicine, such as research from nonmedical fields like psychology, which does not involve any aspect of medical education.
- Non-English language publications.

### Screening and Data Extraction

Before formally determining the inclusion or exclusion of literature, 3 researchers (JZ, SL, and Xin Liu) randomly selected 30 studies for an initial screening to assess the reliability of the screening process. The final calculated Cohen κ value was 0.89, indicating high consistency, and no adjustments were made to the inclusion or exclusion criteria or the researchers involved. In the formal independent screening process, any disagreements were ultimately resolved through intervention by SS. The screening and validation process was completed on April 26, 2025.

To accurately extract data from the included studies, we followed the PRISMA-ScR and created a data extraction form using Microsoft Excel. Two evaluators (JZ and SS) independently completed the form after receiving professional training based on the *Medical Literature Information Retrieval* [29] textbook. Any disagreements between the evaluators were resolved through discussion.

First, we extracted general information from the studies, including the publication year, country, study type, and research objectives. To gain a deeper understanding of the potential applications and challenges of LLMs in medicine, we summarized the key findings and limitations of each study.

Next, the design data extracted consisted of two aspects: (1) general characteristics of LLMs, such as model type, open-source availability, model training (fine-tuning and prompt engineering), and other technologies or tools integrated into LLM-based virtual patients, such as voice assistants and hardware devices, to illustrate the specific design of LLM-based virtual patients; and (2) medical specialty, medical context and tasks, simulated patients, avatars (eg, social robots), participants, and sample sizes to demonstrate the specific application design of LLM-based virtual patients.

Finally, the evaluation data extracted included the evaluation domains (user experience, learning outcomes), evaluation tools (eg, scales and questionnaires), and evaluators (eg, experts) to provide an overview of the overall evaluation details of LLM-based virtual patients use in each study. The data extraction table is provided in Table S2 in Multimedia Appendix 2.

Additionally, to better understand the differences between the studies, we used 2 separate tools for quality assessment (2 authors [WQ and SS] conducted separate evaluations, and any discrepancies in the final scores were addressed through intervention and discussion by a third author [SC]). Medical Education Research Study Quality Instrument [30], a validated tool for evaluating the quality of quantitative medical education research, has a scoring range from 5=lowest quality to 18=highest quality. For qualitative research, we used the QualSyst standard [31], which includes a checklist of 10 criteria for assessing qualitative studies. The score, representing the ratio of obtained points to the maximum possible score, ranges from 0=lowest quality to 1=highest quality. For mixed methods studies, we used both tools simultaneously and reported the scores separately.

For the extracted data, in addition to using the PRISMA-ScR method, we used various other techniques including narrative synthesis, thematic analysis, mapping or data visualization, and descriptive statistical analysis. These methods were used to describe, summarize, and present the application scenarios, research progress, advantages, and limitations of LLM-based virtual patients in medical education in comprehensive formats such as tables, flowcharts, and diagrams.

## Results

### Study Selection

**Figure 1:**
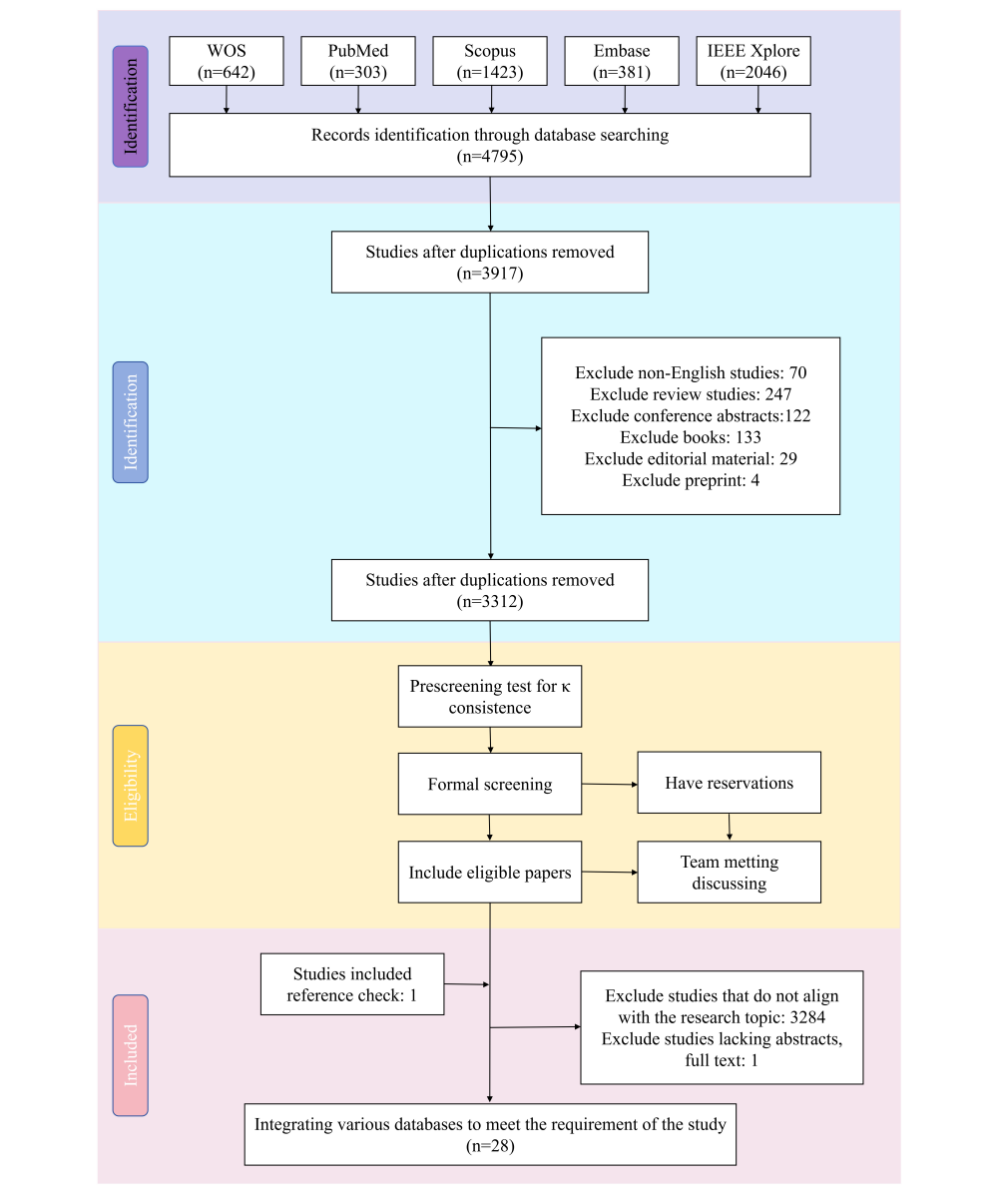
PRISMA (Preferred Reporting Items for Systematic Reviews and Meta-Analyses) flow diagram of study selection. WOS: Web of Science.

A preliminary search across the 5 databases identified a total of 4795 papers. After removing duplicates, 3917 papers remained. Non-English language papers, reviews, conference abstracts, editorials, preprints, and similar publications were excluded, leaving 3312 papers. Three researchers (JZ, SS, and SL) screened the titles and abstracts of these papers and conducted further evaluation, resulting in 27 studies that met the inclusion criteria for this review. An additional study that met all the inclusion criteria was identified through citations in the included papers. Therefore, a total of 28 studies were included in this review. Figure 1 shows the PRISMA (Preferred Reporting Items for Systematic Reviews and Meta-Analyses) flowchart.

### Study Characteristics

The included studies (N=28) were published between 2023 and 2025, with the majority concentrated in 2024 and 2025, accounting for 92.9% (26/28) of the total. This field has attracted widespread attention across various countries and regions, with research conducted in 13 different countries or regions. The top 3 countries with the most studies were the United States (6/28, 21.4%), Germany (5/28, 17.9%), and Japan (3/28, 10.7%). The basic information and quality assessment results for each study can be found in Multimedia Appendix 3.

### Development and Design

In total, 7 studies involved the development and design of LLM-based virtual patient programs or platforms [32-38]. Weisman et al [32] provided a detailed description of the steps taken by their team in developing a GPT-4–based virtual simulated patient and communication training platform. Prior to development, they conducted in-depth interviews to guide design decisions. Other studies provided a more general overview of LLM-based virtual patient applications or platforms. Notably, the virtual patient developed by Shindo et al [36] included a mechanism that identifies and corrects overly detailed initial responses from ChatGPT.

### Practical Applications

#### Overview

In total, 23 studies assessed the actual effectiveness of LLM-based virtual patients, with 2 studies first focusing on design and development before evaluating the practical application of LLM-based virtual patients [32,33]. In contrast, studies evaluating the actual application accounted for over 80% (23/28) of the total, indicating that LLM-based virtual patients in medical education are shifting from the technical development phase to the application phase. This transition highlights the potential anticipated in improving the quality and efficiency of medical education. This is also the primary focus of our study, and we will present a clear and comprehensive overview of the entire process, from research design to application and evaluation, across 5 key aspects.

#### Medical Field and Educational Tasks

These studies cover 13 medical specialties, with the highest number in general medicine (n=9), followed by rheumatology and dentistry, each with 2 studies. All these studies are related to medical training in medical education, specifically examining whether interaction with LLM-based virtual patients can help train key medical skills such as history taking and clinical reasoning. For example, Baugerud et al [39] explored the potential of a ChatGPT-3–based child avatar to train participants in interview skills in the context of suspected abuse. Regarding LLM-simulated patients, all but 2 studies explicitly defined the types of simulated patients used [40,41], as detailed in Table 1.

**Table 1:** Research directions, medical fields, and research content included in large language model–based virtual patient studies.

Reference	Application category	Medical field	Simulated patients
Benfatah et al [42]	Communication skills	Nursing	Patient with respiratory distress
Borg et al [43]	Clinical reasoning	Rheumatology	Patient with rheumatic disease
Brügge et al [20]	Clinical decision-making	Neurology or neurosurgery	Patient with herniated lumbar disc or stroke or meningitis or concussion
Gray et al [44]	N/A^a^	Obstetrics	A mother expecting preterm delivery
Holderried et al [45]	History taking	General medicine	Patient with nausea, weight loss, and chronic fatigue
Holderried et al [21]	History taking	General medicine	Patient with nausea, weight loss, and chronic fatigue
Gutiérrez Maquilón et al [46]	Communication skills	Emergency medicine	A survivor of a traffic accident
Sardesai et al [47]	Anesthesia training	Anesthesiology	Patient with fractured humerus
Yamamoto et al [48]	Interviewing skills	General medicine	Patient with chest pain or abdominal pain or cough or heartburn or fatigue or fever or dizziness or shortness of breath
Aster et al [49]	Empathic expression	Cardiology and emergency medicine	Patient with cardiac conditions
Borg et al [50]	Clinical reasoning	Rheumatology	Patient with rheumatic disease
Cook et al [51]	N/A	Ambulatory medicine	Patient with diabetes or chronic cough
Ko et al [52]	Information gathering skills	Dental	Child survivor of abuse
Öncü et al [53]	Clinical case management	General medicine	Patient with hypertension or brucellosis
Rädel-Ablass et al [54]	History taking	General medicine	Patient with brain hemorrhage
Wang et al [55]	History taking	Gastroenterology	Patient with inflammatory bowel disease
Or et al [56]	History taking	Dental	Patient with dental conditions
Cook [18]	N/A	General medicine	Patient with chronic cough or type 2 diabetes
Yi and Kim [33]	History taking	Urology	Patient with urinary problem
Abou Karam [40]	N/A	General medicine	N/A
Weisman et al [32]	Communication skills	General medicine	Patient with abnormal mammogram results
Baugerud et al [39]	Interviewing skills	Psychology	Child survivor of sexual or physical abuse
Liu et al [41]	N/A	General medicine	N/A

^a^N/A: not applicable.

#### LLM Strategy and Prompting

OpenAI’s GPT series models are the most frequently used, with 95.7% (22/23) of the studies incorporating them, the majority using more advanced versions, namely, ChatGPT-3.5 or higher. Other LLMs used include HyperCLOVA. Detailed information is provided in Table 2.

**Table 2:** Information on the use of models in large language model–based virtual patients, language, fine-tuning, and prompts.

LLMs	Language	Fine-tuning	Prompt	Reference
ChatGPT (not specified)	Not specified	No	Scenario	[42]
GPT-3.5-turbo	English	No	Scenario, behavioral, emotional simulation	[43]
GPT-3.5	German	No	Scenario, behavioral, communication style, feedback	[20]
GPT-3.5	English	No	Scenario, communication style, emotional simulation	[44]
GPT-4	German	No	Scenario, behavioral, feedback	[45]
GPT-3.5-turbo	German	No	Scenario, behavioral	[21]
GPT-3.5-turbo	German	No	Scenario, behavioral, communication style, emotional simulation	[46]
Not specified	English	Custom knowledge bank	Not specified	[47]
GPT-4-turbo	Japanese	No	Scenario, emotional simulation	[48]
GPT-3.5	German	No	Scenario, behavioral, communication style	[49]
GPT-3.5-turbo	English	No	Scenario, behavioral, emotional simulation	[50]
GPT-3.5-turbo or 4-turbo	English	No	Scenario, behavioral, communication style, emotional simulation, feedback	[51]
GPT-3	Norwegian	No	Scenario	[52]
GPT-4o	English	No	Scenario	[53]
GPT-4	Not specified	No	Scenario, behavioral, communication style	[54]
GPT-4	English or Chinese	No	Scenario, behavioral, communication style, emotional simulation	[55]
GPT-3.5 Instruct	English	No	Not specified	[56]
HyperCLOVA	Korean	Scripts of medical interviews	Not specified	[33]
GPT-3.5	Not specified	No	Not specified	[40]
GPT-4	English	No	Scenario, behavioral, emotional simulation, feedback	[32]
GPT-3.5-turbo or 4	English	No	Scenario, behavioral, communication style, emotional simulation	[18]
GPT-3	Not specified	741 mock interviews	Not specified	[39]
GPT-3.5-turbo	Not specified	No	Not specified	[41]

In the research process, to better adapt the LLM to specific application needs and improve its performance in simulating patients, some researchers fine-tuned the models. Fine-tuning involves updating the model’s weights using smaller, domain-specific corpora. In total, 13% (3/23) of the studies involved fine-tuning the LLMs used for simulated patients [33,39,47]. For instance, Baugerud et al [39] fine-tuned their model using 741 mock interviews sourced from a forensic interview training program.

To ensure the desired interaction between participants and LLM-simulated patients, carefully designed prompts were used to elicit the required responses from the LLMs. In total, 69.6% (16/23) of the studies described or provided the full prompts used in their research, of which 26.1% (6/23) of the studies used prompt engineering to iteratively optimize the prompts [20,21,32,45,54,55]. A notable example is the study by Wang et al [55], where they conducted multiple preliminary tests to standardize the evaluation of the model’s responses, generated a list of questions, and then adjusted the prompts until the LLM-based virtual patients performed optimally. Only then, did they finalize the prompt version. A deeper analysis of the 16 studies that described or provided full prompts revealed that the prompts could be classified into five types: (1) contextual prompts: providing background information and setting the interaction scenario, (2) behavioral prompts: restricting or guiding the model’s responses, (3) communication style prompts: adjusting the language style or changing the mode of communication, (4) emotional simulation prompts: incorporating specific emotional responses into the answers, and (5) feedback prompts: offering personalized suggestions based on user performance. The types of prompts used in each study are provided in Table 2, and typical examples of each prompt type are presented in Table S1 in Multimedia Appendix 4.

#### Instructional Design Features

A summary of the scenarios presented in each study revealed that the most frequently reported scenario was taking a history inventory with an LLM-based virtual patient (20/23, 87%). Each study’s scenarios were reviewed based on the Calgary-Cambridge model of medical communication steps [57], which include (1) gathering information from the patient, (2) building a relationship with the patient, and (3) explaining and planning. Excluding 3 studies [18,32,40], the remaining 87% (20/23) of the studies reported communication skills related to gathering information from the patient. No studies involved building a relationship with the patient or explaining and planning. All studies focused solely on verbal communication skills, without addressing nonverbal behaviors such as gestures or nodding.

In total, 21.7% (5/23) of the studies focused on aspects aimed at improving learning outcomes [20,32,45,51,52], specifically the accuracy and usefulness of feedback automatically generated by LLM-based virtual patients. For instance, Brügge et al [20] demonstrated that the intervention group receiving AI-generated personalized feedback performed better in clinical decision-making (CDM) than the control group, supporting the effectiveness of feedback. Additionally, 4.3% (1/23) of the study incorporated an additional learning activity, namely, a group-based follow-up workshop [43]. In this workshop, students could discuss and share any issues encountered during the LLM-based virtual patient exercises. Such workshops facilitated better learning outcomes after practicing with LLM-based virtual patients.

#### Technological Design Features

LLM-based virtual patients are presented through integrated technological ecosystems such as the web, hardware, platforms, software, or applications (Table 3). The most commonly used platform is OpenAI’s public web user interface, used in 7 studies, followed by laptops and social robots, each used in 2 studies. Additionally, to achieve more immersive interactions with LLM-based virtual patients, some studies incorporated advanced technological ecosystems such as mixed reality (MR) and virtual reality (VR). For example, Gutiérrez Maquilón et al [46] examined the integration of GPT-based AI into an MR virtual patient application for communication training of emergency medical services personnel. The system delivered an immersive, sensorially rich MR environment that closely simulated real-world emergency scenarios, maximizing ecological validity.

**Table 3:** Technology ecosystem, interaction modality, and auxiliary tools in the application research of large language model–based virtual patients.

Technology ecosystem			Interaction modality		Auxiliary tools		Reference
**Web**							
	OpenAI’s public web UI^a^	Text		No		[20]	
	OpenAI’s public web UI	Text		No		[44]	
	OpenAI’s public web UI	Text		No		[49]	
	OpenAI’s public web UI	Text		No		[55]	
	OpenAI’s public web UI	Text		No		[18]	
	OpenAI’s public web UI	Text		No		[33]	
	OpenAI’s public web UI	Text		No		[41]	
	Self-developed web interface	Text		No		[21]	
**Hardware**							
	Laptops	Text		No		[42]	
	Social robot	Robot+voice		Furhat software development kit		[43]	
	Laptops	Text		No		[45]	
	MR^b^	3D avatar rendering+voice		Microsoft LifeChat LX-3000 headset, OpenAI Whisper, ElevenLabs		[46]	
	Social robot	Robot+voice		Furhat software development kit		[50]	
	Tablet	Voice		No		[53]	
	VR^c^	3D avatar rendering+voice		IBM Watson services		[39]	
**Platform**							
	ConvAI	2D avatar+text or voice		No		[47]	
	Miibo or LINE Corporation	Text		No		[48]	
	Guided Conversation Designer	Text		No		[54]	
	Vercel	Text		No		[56]	
**Software or program**							
	Python	Text		No		[51]	
	Hyperskill	Voice		Hyperskill		[32]	
**Other**							
	Not specified	Static avatar+text		No		[52]	
	N/A^d^	N/A		N/A		[40]	

^a^UI: user interface.

^b^MR: mixed reality.

^c^VR: virtual reality.

^d^N/A: not applicable.

Regarding interaction modes, the majority of studies used natural language, including text (n=14) and voice (n=7), with 1 study supporting both modes. In contrast, the languages used in the studies were more diverse, encompassing 5 different languages (Table 2). One study tested the impact of Chinese and English on LLM-based virtual patient performance, finding no significant performance differences between the test groups using different language combinations [55].

Furthermore, only 26.1% (6/23) of the studies used patient avatars, 4 of which were virtual avatars [39,46,47,52], and 2 were physical embodiments, that is, social robots. In total, 2 studies provided details on the creation and presentation of virtual avatars, both using the Unity game engine for 3D rendering of human models or virtual avatars, which were then presented to participants using head-mounted displays [39,46]. Details are provided in Table 3.

To enhance the realism of virtual patients and improve the user experience, 21.7% (5/23) of the studies used auxiliary tools for LLM-based virtual patients. These tools can be categorized into voice modules, transcription modules, and emotional visualization modules (details and categorization of tools are provided in Table S2 in Multimedia Appendix 4). A notable example is 2 studies of Borg et al [43,50], where LLM-based virtual patients were presented through social robots, supplemented by the use of FurhatSDK. This setup allowed for voice interaction, as well as the display of subtle facial expressions and emotions, making the virtual patients more anthropomorphic [43,50].

#### Evaluation Features

Among the 23 studies, a total of 398 participants were clearly identified as experimental participants interacting with LLM-based virtual patients, including various groups such as medical students, clinicians, and emergency medical personnel. Medical students comprised 71.6% (285/398) of the participants, while medical educators made up only 0.5% (2/398).

Two methods were used to evaluate the practical application of LLM-based virtual patients: assessing the user experience of LLM-based virtual patients (23 studies) and evaluating the learning outcomes facilitated by LLM-based virtual patients (5 studies) [20,48,49,52,53]. The characteristics of these 2 types of assessments, including the measurement domains, tools, evaluators, and results, are provided in Multimedia Appendix 5.

Based on Nielsen’s usability concepts [58] and existing literature on the assessment criteria for standardized patients and virtual patients [25,59], we reviewed the user evaluations of LLM-based virtual patients used in these studies. The evaluation standards included (1) technological design, (2) realism of simulation, and (3) practicality of learning. In total, 47.8% (11/23) of the studies commonly used at least 1 of the following standards to assess technological design: (1) usability (overall experience or perception after use), (2) satisfaction (acceptance), and (3) errors (technical issues). Regarding the realism of simulation, 60.9% (14/23) of the studies assessed it by asking about authenticity or contextualization. Authenticity (the degree to which LLM-based virtual patients resemble real patients) was the most focused-on area (14/14, 100%). In total, 21.4% (3/14) of the studies assessed contextualization (how closely the simulation resembles real-world scenarios). A total of 39.1% (9/23) of the studies evaluated users’ perceptions of the practicality of LLM-based virtual patients in learning, specifically in terms of the learning process (how much they helped achieve target skills) or feedback quality (usefulness or appropriateness of the provided feedback).

For the measurement tools, 3 validated questionnaires were used to assess usability in the technological characteristics [21,46]; 1 validated questionnaire was used to assess authenticity in the simulation realism [50]. Among the self-developed evaluation tools, 3 studies validated and reviewed their scales or questionnaires, which somewhat enhanced the validity, reliability, and applicability of these self-created assessment tools [33,51,54]. Across the 23 studies that evaluated user experience with LLM-based virtual patients, a total of 22 different assessment tools were used. Among these, 18 studies used self-assessment tools completed by users, while 4 studies used expert assessments conducted by clinicians, medical professors, or other domain experts.

Although these studies applied to various skills training (Table 1), only 21.7% (5/23) of the studies assessed learning outcomes in terms of objective skill measurements. In these studies, the researchers measured changes in learners’ skills, such as CDM, and empathetic expression in specific scenarios. One study used a self-created tool for objective skill measurement [53], while the others used validated or reliable tools. For example, the Clinical Reasoning Indicator-History Taking Inventory was used to measure clinical reasoning skills [20]. Of the 5 studies evaluating learning outcomes, 4 used expert assessment, and 1 used self-assessment.

Quality assessment revealed heterogeneity and frequent inconsistencies in the study designs and evaluations, making it challenging to assess the performance of LLM-based virtual patients. Therefore, we provided a general overview of the research findings. Information on the tasks, performance or results, sample sizes, clinical validation methods, and participant demographics for each study can be found in Multimedia Appendix 6. Among these studies, 2 were controlled experiments. The remaining studies were observational in nature. Overall, the included studies demonstrated a positive attitude toward the application of LLM-based virtual patients.

## Discussion

### Principal Findings

Our review indicates that the application of LLM-based virtual patients has gained considerable momentum in recent years, with many research teams developing diverse and innovative applications. However, the heterogeneity in study designs, evaluation standards, learning outcomes, and their measurements limits the ability to make direct comparisons and draw definitive conclusions, indicating that future research has much ground to cover.

### LLMs for Simulated Patients

In these studies, the primary LLM used is the ChatGPT series, indicating that current research on LLM-based virtual patients heavily relies on proprietary models. ChatGPT is a closed-source proprietary model, which raises significant open science issues related to transparency and reproducibility [60,61]. Different OpenAI models, in particular, exhibit notable differences; yet, the source of these discrepancies remains opaque, as OpenAI’s closed-source policy makes testing and evaluation impossible. Additionally, OpenAI (and similar systems) continuously updates its models, meaning that research conducted using ChatGPT today may not be directly replicable, or even reproducible, within the next 6 months [62]. This presents challenges to the reproducibility of LLM-based virtual patient research outcomes. In contrast to closed-source (or proprietary) models, open-source models are not affected by undisclosed updates and can be fully deployed locally, avoiding some clinical data privacy issues associated with closed-source models [63]. Furthermore, using open-source LLMs is crucial for reproducibility. With open-source LLMs, researchers can examine the internal structure of the model to understand how it works, customize the code, and flag errors [64]. These details include adjustable parameters and the data on which the model is trained. Currently, many high-performance open-source LLMs, such as DeepSeek and LLaMA, have emerged, demonstrating strong capabilities in specific domains [65]. Exploring the use of these models could reduce reliance on a single model, greatly improving the universality and reproducibility of research.

To enhance the scientific rigor and scalability of LLM-based simulated patients, the research community must adopt more controlled methodologies. Among studies using ChatGPT, only 2 mentioned setting the “temperature” hyperparameter [49,51], with one of them exploring whether the temperature affects LLM-based virtual patients’ performance. The results indicated that there were no significant differences in performance at different temperatures, but further research is needed to validate this outcome. Temperature is a frequently modified hyperparameter that controls the randomness of the model’s predictions [66]. Some researchers believe that temperature features will play a crucial role in the application of generative AI in medical services, potentially enabling more accurate, empathetic, or creative interactions between AI and health care stakeholders [67]. Currently, there is limited research on the hyperparameters used for LLMs simulating virtual patients. Besides temperature, other parameters, such as the 2 “repetition penalties,” which reduce token repetition and may make responses more diverse, remain unexplored in the context of LLM-based virtual patients. Whether these parameters contribute to more realistic simulations of virtual patients is yet to be determined. Additionally, researchers must address issues related to backend model updates and random factors in the sampling process to ensure the reliability of results [68].

### Application of LLM-Based Virtual Patients

The design features of virtual patients, such as interactivity, play an important role in enhancing clinical reasoning skills [69]. Compared to less interactive approaches, highly interactive virtual patients allow educators to better assess students’ clinical reasoning skills by directly observing their abilities [70]. Leveraging advanced technological ecosystems, such as social robots, MR, VR, and auxiliary tools, LLM-based virtual patients can achieve higher levels of interactivity (eg, speech, movement, and eye contact), creating more authentic simulated encounters with potential to enhance learning [71,72]. However, only a minority of studies addressed this dimension.

Despite the potential for realistic simulated interactions, several technical barriers hinder the use of LLM-based virtual patients at a high level. First, LLMs face challenges in emotional understanding and perception. Although LLMs possess subtle capabilities in understanding and managing emotions, they are inefficient in using emotions to facilitate thinking [73]. This results in issues like unrealistic emotional expression and incongruent emotional responses in LLM-based virtual patients. Moreover, training LLMs with multimodal datasets—especially those incorporating speech and video data—can improve the model’s understanding of a patient’s emotional and contextual state, enhancing the naturalness and accuracy of dialogues. However, incorporating these data modalities raises significant privacy concerns, as speech and video data not only threaten patient privacy but also the privacy of clinicians [74], limiting the use of multimodal datasets. Additionally, the embodiment of virtual patients presents challenges. To achieve realistic interactions and an immersive experience, LLM-based virtual patients typically rely on social robots, VR, and similar hardware. Currently, most robots are designed to express basic emotions and lack sufficient smooth and accurate facial movements, such as eye movements, blinking, eyebrow movements, and particularly lip movements [75,76], hindering more realistic simulation of patient reactions and symptom presentation. VR hardware also faces challenges, including high demands for computational resources and persistent issues with rendering delays [77], which can significantly impact the performance of virtual patients, causing interactions to be sluggish and unnatural, potentially lowering training and learning outcomes.

At present, the application of LLM-based virtual patients primarily focuses on the users, such as medical students and interns, while the core figures driving medical education—such as teachers—have received less attention. Research by Montenegro-Rueda et al [78] shows that integrating ChatGPT into the educational environment can positively impact the teaching process, but its successful implementation depends on the proficiency of the educators, making adequate teacher training key to effective use. Advanced technologies may enhance learning outcomes, but without well-designed curricula or teaching strategies, specific learning results cannot be guaranteed [25]. Lövquist et al [79] argue that establishing and maintaining close relationships between educators, clinicians, and developers are crucial for the development of effective, reliable, and useful VR-based medical training and assessment systems. Similarly, for the development of LLM-based virtual patient medical training and assessment systems, educators’ involvement is essential. Identifying teaching strategies, such as how educators demonstrate the use of virtual patients, explain the medical simulation setup, and provide feedback, plays a significant role in shaping students’ learning outcomes, academic performance, and overall development [80]. Furthermore, this involvement fosters positive teacher-student relationships, which can be reflected in students’ focus and interest in the course content [81]. From the student perspective, experiencing teacher support helps them feel a sense of belonging in the classroom, which enhances their emotional learning, such as their attitude toward the content, thus strengthening their effective learning [81-83]. Additionally, teaching design, often overlooked, is a key factor in optimizing the use of technology and determining its effectiveness. While LLM-based virtual patients indeed have unique and optimal characteristics for medical communication training, their use must be guided by carefully designed teaching interventions to ensure effectiveness. For instance, designing collaborative pair activities or group discussions after interactions with virtual patients can bring added benefits, including increased interactivity, better use of the virtual patient platform, and improved clinical reasoning training [43,84].

Communication is a complex phenomenon that involves not only verbal language but also various nonverbal channels and responses [85]. Nonverbal communication includes conveying information through body signals, such as eye contact, facial expressions, gestures, and acoustic cues (paralanguage) [86]. However, the reviewed studies mostly focused on verbal communication, with limited attention to nonverbal behaviors. These nonverbal elements often carry as much, if not more, information than verbal communication itself. For instance, in interactions with patients, doctors primarily rely on facial expressions, body language, vocal tone, and other subtle cues to interpret meaning and make clinical decisions [87]. Some studies have shown that medical students’ communication skills, particularly in nonverbal communication and empathy toward patients, are insufficient [88-90], highlighting the need to focus on improving learners’ nonverbal communication skills. To train nonverbal communication skills using LLM-based virtual patients, more advanced LLMs, such as multimodal LLMs, must be used, in combination with more advanced technological ecosystems. However, limited by technological and cost-effectiveness issues, especially technical limitations like system failures, language processing challenges, and system overloads [91], as well as the inability of virtual patients to fully simulate real patient responses, achieving the goal of training nonverbal communication skills remains challenging.

To enhance the realism of LLM-based virtual patients, fine-tuning LLMs with training data relevant to target outcomes is necessary. Bui et al [92] fine-tuned 3 open-source models using existing datasets, data scraped from Vietnamese medical online forums, and data extracted from Vietnamese medical textbooks. The results showed that the fine-tuned models performed better than their base versions on evaluation metrics such as BertScore, Rouge-L, and the “LLM as Judge” method, confirming the effectiveness of the fine-tuning process. Currently, only a few studies mention fine-tuning, and no detailed investigations have been conducted. Future research should explore whether fine-tuning LLM-based virtual patients using different specific types of data, such as medical dialogues, can optimize their performance. Previous studies have fine-tuned LLMs using doctor-patient dialogue datasets, showing significant improvements in the model’s ability to understand patient needs and provide targeted suggestions [93]. Furthermore, only a few studies have explicitly addressed prompt engineering in the design of LLM-based virtual patient prompts. By optimizing the input structure, prompt engineering plays a crucial role in refining AI and LLM outputs [94]. Modifying and optimizing prompts to make them more specific lead to more accurate and focused LLM outputs [95], thus improving the performance of LLM-based virtual patients and enabling more realistic and accurate simulated education.

### Evaluation of LLM-Based Virtual Patients

The design and evaluation of the 23 studies on the practical application of LLM-based virtual patients are heterogeneous and often inconsistent, making it difficult to accurately assess the task performance and application effectiveness of LLM-based virtual patients and masking the potential of LLM-based virtual patients in medical education. Particularly in terms of evaluation, only a few studies used validated tools to assess LLM-based virtual patients, indicating a lack of standardization in the evaluation process. Furthermore, evaluations have largely focused on subjective indicators—users’ experiences—which means that the heterogeneity of these outcome measures may prevent cross-study comparisons. While these studies show enthusiasm for the use of LLM-based virtual patients in medical training, whether they can be further applied to medical education needs careful consideration. Due to the lack of standardization, LLM-driven virtual standardized patient training is unlikely to be used as part of summative clinical examinations or assessments of learner communication skills. The limitations of current evaluation standards highlight the need for broader evaluations of simulation robustness.

Currently, several effective and reliable evaluation methods or frameworks could be considered for future research. To assess user experience, tools like the Subjective Assessment of Speech System Interfaces (SASSI) questionnaire [96] and Witmer’s Presence Questionnaire [97] could be used. The SASSI questionnaire is an effective, reliable, and sensitive measure of users’ subjective experience with speech recognition systems, including dimensions such as system response accuracy, likability, cognitive demand, annoyance, habitability, and speed. It includes 39 Likert items, scored from 1=strongly disagree to 7=strongly agree. Using this questionnaire to assess the usability of LLM-based virtual patients in voice interaction could provide insights into speech recognition accuracy, interaction smoothness, and users’ experiences. However, the SASSI has been used only in limited speech recognition systems. Witmer’s Presence Questionnaire, consisting of 22 self-report items, each with a 7-point Likert scale, assesses the sense of immersion in virtual environments, with higher scores indicating stronger immersion.

From an educational training perspective, models like Kirkpatrick’s 4-level training evaluation model and Kolb’s experiential learning theory could be introduced. Kirkpatrick’s model, introduced in 1959, is one of the most widely used and well-known frameworks for evaluating training and development programs. It has 4 levels: reaction, learning, behavior, and results. Due to its robustness, adaptability, and applicability, using this model could lead to more effective evaluation of training outcomes [98]. Since only 5 (17.9%) studies measured learning outcomes, applying this model to assess the training effectiveness of LLM-based virtual patients in medical training could enable researchers to provide a more comprehensive demonstration of virtual patient systems’ impact on medical education, showcasing learners’ performance after virtual patient training and analyzing improvements in knowledge mastery, skill application, and CDM, rather than merely focusing on learners’ immediate reactions or academic performance. Kolb’s experiential learning theory emphasizes the process of learning through experience as an integrated cycle of 4 stages: concrete experience, reflective observation, abstract conceptualization, and active experimentation. Each stage is interrelated, guiding learners from direct experience to critical reflection, conceptual understanding, and the application of new knowledge [99]. Using this framework to guide the evaluation of LLM-based virtual patient systems could not only provide students with an immersive learning experience but also help medical students combine theory with practice through continuous feedback, reflection, and experimentation. Additionally, frameworks for automated interaction assessment and AI-structured clinical examinations to assess LLM performance in clinical tasks could also be adapted [100,101].

Only 5 studies involved the measurement of objective skills, making it unclear how effective LLM-based virtual patients are in training users’ skills. This not only reflects the intent of clinical educators but also highlights the characteristics of the simulated training environment, which often faces time constraints and limited resources, making long-term sustainability difficult [102]. Overall, the current evaluation practices for LLM-based virtual patients in communication training lack rigor. More controlled approaches are needed to improve scalability and scientific rigor, such as using validated tools and systematically examining changes in student behavior or clinical outcomes, rather than simply focusing on students’ attitudes or performance in the simulated environment. Additionally, researchers could reduce the limitations associated with self-reported data by collecting psychophysiological data from digital sensors (eg, electroencephalography, extreme energy ratio, and heart rate) during students’ interactions with virtual patients and providing real-time feedback [103]. In conclusion, the insufficient standardized assessment of learning outcomes means we must remain cautious in judging the practical value of LLM-based virtual patients, though all 23 studies show positive user attitudes toward LLM-based virtual patients. While this attitude does not equate to scientifically validated educational effectiveness, it can help enhance learners’ motivation, undeniably indicating the promising potential of LLM-based virtual patients in medical training.

Privacy and security, key aspects often not addressed or discussed in the studies, are important considerations in LLM-based virtual patient research. Most of the studies used cloud-based LLMs, but cloud-based LLMs typically require users to upload explicit requests during inference, which inevitably raises concerns about data security and user privacy [104-107]. Specifically, the process of guiding LLMs to simulate patients through prompts inevitably includes patient-related information. The popular privacy protection method for LLMs is to encrypt user medical requests to prevent LLM service providers’ servers from accessing private user data. Common methods, such as solid-state encryption technology [108], significantly mitigate the risk of LLM operators or potential attackers leaking or misusing patient data for commercial or other purposes [109]. While these privacy protection methods are effective, challenges remain for medical LLMs, such as resource consumption and potential impacts on model accuracy and reliability [110]. A new method, adaptive compressed-based privacy-preserving LLM, has been proposed, which avoids the aforementioned issues while demonstrating strong privacy protection capabilities and high response accuracy [110]. Local deployment of LLMs is also a reliable method for addressing data leakage and privacy concerns. This approach ensures that user data stay within the organization, significantly enhancing data security and privacy protection [111]. Researchers have proposed an innovative compact LLM framework for local deployment of electronic health record data, which not only addresses privacy concerns in medical environments but also overcomes challenges related to limited computational resources [112]. Furthermore, from a patient data perspective, using synthetic patient data can help resolve privacy issues [113]. Synthetic data do not pose the same privacy concerns as real patient data because they are not linked to any specific individual [114].

### The Ethics of Using LLM-Based Virtual Patients

The use of LLM-based virtual patients in medical education involves several ethical dimensions, including data ownership and consent for use, data representativeness and bias, and privacy [115]. These dimensions reflect the relationships, responsibilities, and moral obligations between virtual patients as a technological tool and actual patients, health care professionals, and educators.

#### Data Ownership and Consent for Use

Using LLM-based virtual patients with patient data raises core questions about ownership, consent, and anonymization. When fine-tuning models or supplying prompts, patients may need explicit consent or at minimum clear notice that their data are used. Safeguarding informed consent and data rights is central to the ethics of virtual patient simulations.

#### Data Representativeness and Bias

LLMs may present potential algorithmic biases that lead to discriminatory behaviors and stereotypes, potentially resulting in unfair treatment of certain groups [116-119]. If these biases are not identified and corrected in a timely manner, virtual patients may contribute to incorrect diagnoses or treatment plans in certain populations, thus exacerbating inequalities in health care services. It is imperative that researchers and developers of LLM-based virtual patients proactively address these biases to prevent harmful consequences and ensure equitable health care training environments.

#### Privacy

The application of LLM-based virtual patients may involve the processing of real patient-related data. Even when using synthetic or virtual patient data, it is crucial to ensure that the data are deidentified, anonymized, and fully protected to prevent potential personal information leaks. Adequate safeguards must be implemented to protect patient privacy and ensure that virtual patient data are handled ethically and securely.

### Situation of LLM-Based Virtual Patients

**Figure 2:**
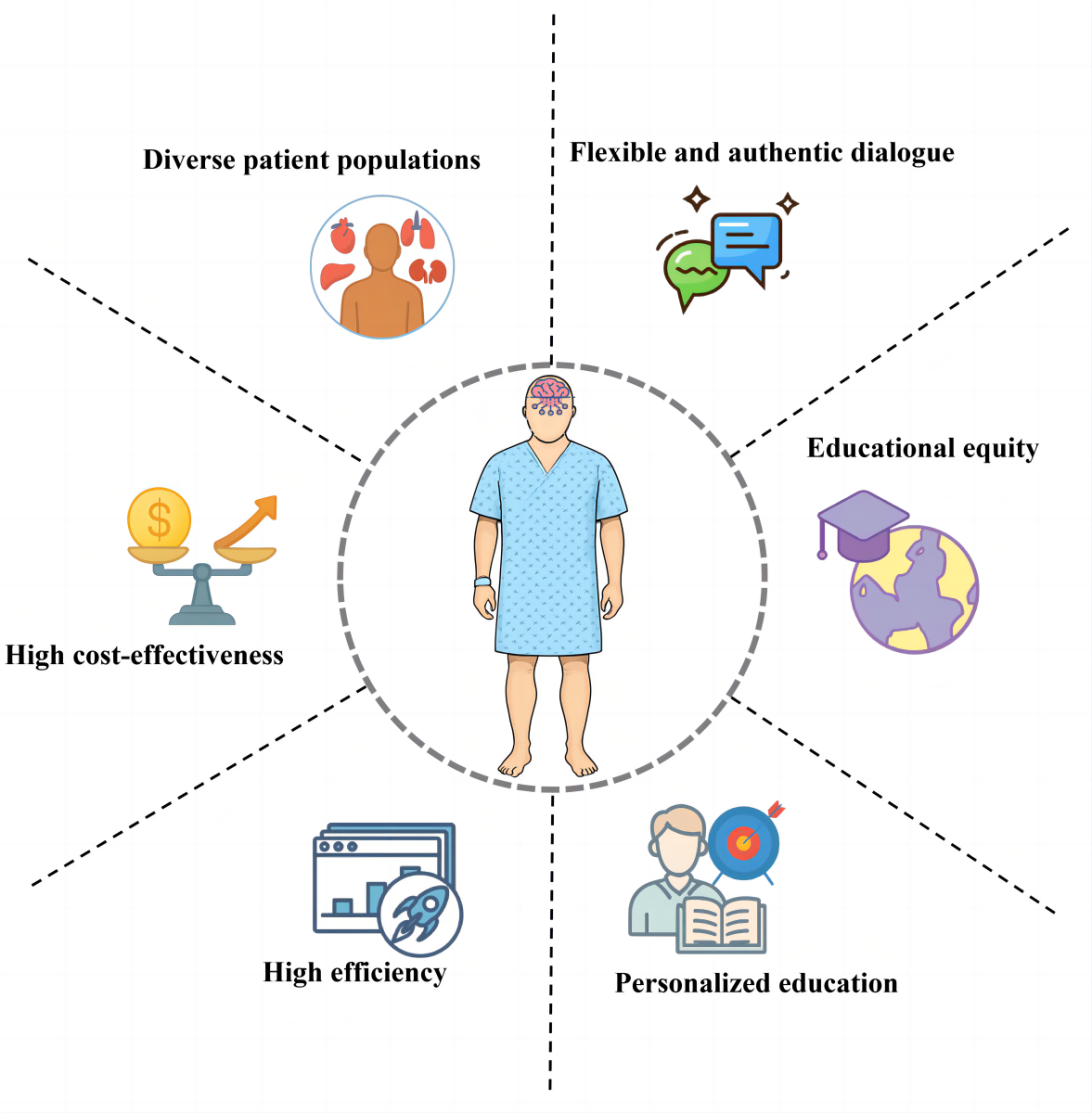
Advantages of using large language model–based virtual patients in medical education and training.

Integrating AI into medical education is crucial for equipping health care professionals with the key skills needed to provide optimal patient care in the future [120], and the use of LLM-based virtual patients undoubtedly aligns with this trend. Compared to most existing virtual patients, LLM-based virtual patients demonstrate unscripted, responsive dialogues that exhibit realism and flexibility. This realism is advantageous for training, assessment, and research on shared decision-making [121-124] and other management reasoning processes [125-127]. LLMs can simulate a diverse range of patients. For rare diseases, medical students often find it difficult to encounter real patients experiencing these conditions during clinical rotations. LLM-based virtual patients offer high cost-efficiency, such as reducing the resources required for interaction with real patients and specialized facilities. Through this LLM-based approach, thousands of preference-sensitive virtual patients can be created with greater efficiency and even higher realism than current labor-intensive methods. Each virtual patient can be “created” as a single document page, with different variants added by changing just a few sentences [51]. Furthermore, the application of LLM-based virtual patients can help mitigate educational inequities. AI-driven simulations can be accessed by an unlimited number of students across different geographic locations. The system enables anyone to train at any time, unrestricted by time or spatial limitations, thereby democratizing access to high-quality educational experiences. The advantages of LLM-based virtual patients are shown in Figure 2.

Although LLM-based virtual patients offer numerous advantages, they should be seen as a useful and cost-effective supplementary tool rather than a replacement for real-life interactions. Their greatest limitation lies in their inability to handle nonverbal communication, which is crucial for developing important skills such as empathy communication. Research has shown that while medical students interact with virtual patients with empathy, these interactions, both quantitatively and qualitatively, are insufficient to replace real-life interactions, such as with standardized patients [128]. In conclusion, while the application of LLM-based virtual patients holds great promise, integrating them into medical education is inevitable. However, educators must remain vigilant and consider both the positive and negative impacts that this integration may bring [129].

### Future Development Directions

The rapid development of LLMs has led to the emergence of many high-performance models. Researching the performance of other models in patient simulation can better address the diverse needs of medical training, technological iterations, and security issues. Using representative and diverse samples, such as those from different geographic regions, cultures, and backgrounds, will help ensure the broad applicability and accuracy of the results. Additionally, creating diverse scenarios and establishing standardized evaluation methods to study and assess LLM-based virtual patients are equally important.

The realism of LLM-based virtual patients remains a key area for improvement. Compared to using a single model, collaborative multimodel systems have demonstrated superior performance [130]. Exploring the potential of multiple LLMs working together to reduce errors and enhance realism in simulated patients holds promise. When combined with more advanced technological ecosystems, such as social robots, augmented reality, VR, or MR, these systems can facilitate multisensory interactions, offering significant benefits for medical skills training. Furthermore, further exploration of prompt engineering related to simulating virtual patients can play a critical role in improving the realism of LLM-based virtual patients’ performance.

Currently, the research on LLM-based virtual patients has overlooked safety concerns, particularly data privacy and protection. Using secure local LLMs can reduce data privacy risks, although it requires additional computational resources. Methods for privacy protection still need to be explored and developed in future research.

**Figure 3:**
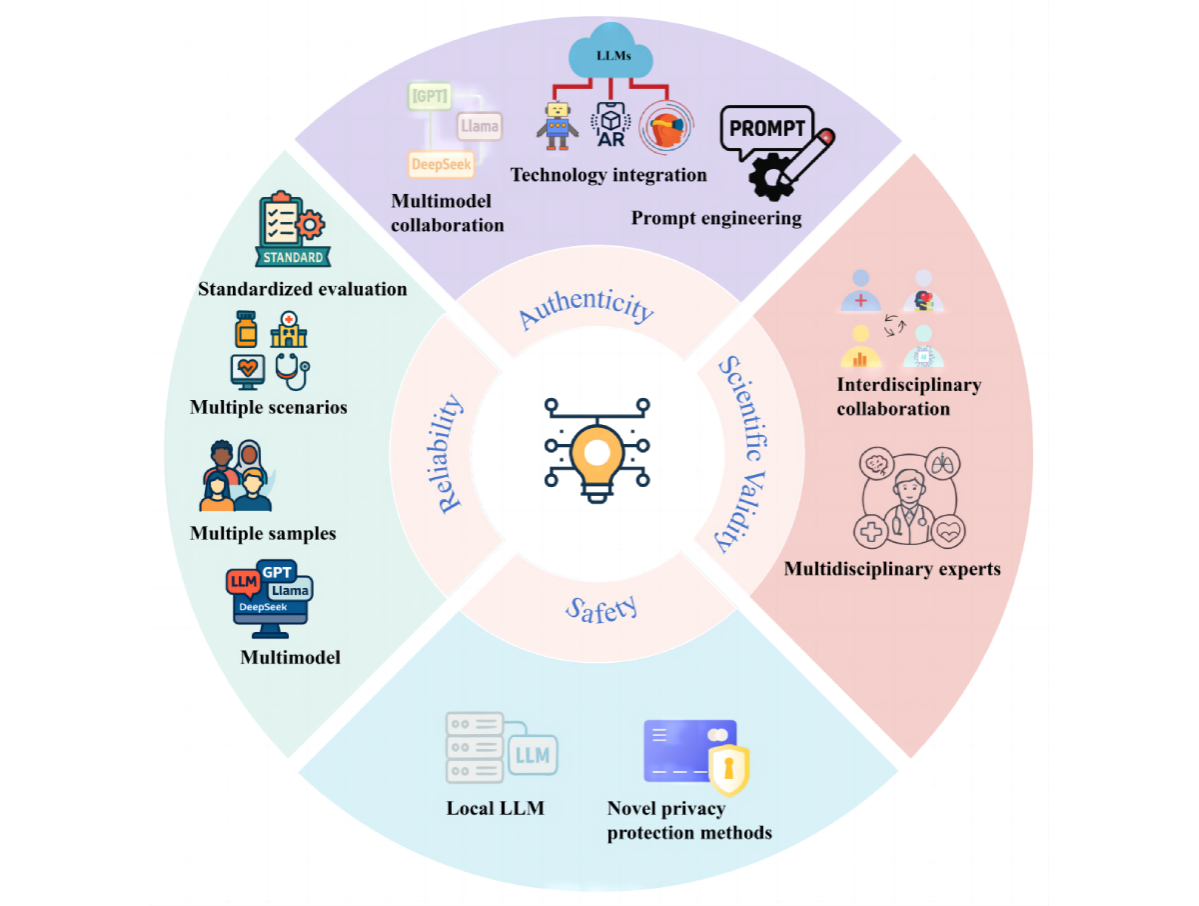
Future development directions in LLM-based virtual patient research. LLM: large language model.

Finally, ensuring the scientific rigor of LLM-based virtual patient design remains an area of further study. Early interprofessional education training has the potential to enhance leadership, collaboration, and communication among health care teams, ultimately improving patient safety [131]. Incorporating the knowledge and experience of multidisciplinary medical experts (such as clinicians, pharmacists, and rehabilitation specialists) to optimize LLM-based virtual patients will allow for more comprehensive and scientific applications, achieving the goal of interprofessional education training for users. Moreover, collaboration among interdisciplinary teams should also be explored. Integrating expertise from medical professionals, data scientists, AI researchers, psychologists, and other fields in the design and development of LLM-based virtual patients will significantly enhance their scientific foundation. The 4 future research directions are illustrated in Figure 3.

### Strengths and Limitations

To the best of our knowledge, this is the first comprehensive review focused on LLMs simulating virtual patients. We have summarized the entire process of using LLM-simulated patients for medical training, covering aspects from experimental design to outcome evaluation. This review highlights the development, design, and application processes, providing valuable references for the higher-quality, more effective, and scientific application of LLM-based virtual patients in medical education.

However, this study has several limitations. First, our review was limited to English-language literature, potentially overlooking high-quality research published in other languages. Second, the inclusion of studies relied on the subjective judgment of the researchers, which may introduce selection bias. Additionally, as research on LLM-based virtual patients is still in its early stages, the limited number of relevant studies may affect the comprehensiveness and representativeness of the analysis.

### Conclusions

This scoping review adopts a rigorous methodology to summarize and discuss the current state of applications of LLM-based virtual patients in medical education. The findings indicate that research on LLM-based virtual patients has gradually increased over the past 2 years. They provide learners with opportunities to repeatedly practice communication skills and receive timely, appropriate feedback, offering significant economic benefits. As a result, they hold promising prospects in delivering effective medical skills training. However, further improvements are needed in areas such as research design, model implementation, humanization, privacy and security, and evaluation criteria. Additionally, it is important to clarify that LLM-based virtual patients should serve as a valuable supplement to traditional simulation-based education rather than a replacement. Future research is essential to further investigate their reliability, authenticity, safety, and scientific rigor.

## Data Availability

The datasets generated and analyzed during this study are available from the corresponding author upon reasonable request.

## Appendix

Multimedia Appendix 1 PRISMA-ScR checklist.

Multimedia Appendix 2 Search strategy and data extraction form.

Multimedia Appendix 3 Basic information for each study.

Multimedia Appendix 4 Typical examples of each prompt and auxiliary tool categories and descriptions.

Multimedia Appendix 5 Evaluation characteristics of large language model–based virtual patients.

Multimedia Appendix 6 Summary of large language model–based virtual patient applications in medical training.

## Abbreviations

AI: artificial intelligence
CDM: clinical decision-making
LLM: large language model
MR: mixed reality
PRISMA: Preferred Reporting Items for Systematic Reviews and Meta-Analyses
PRISMA-ScR: Preferred Reporting Items for Systematic Reviews and Meta-Analyses extension for Scoping Reviews
SASSI: Subjective Assessment of Speech System Interfaces
VR: virtual reality
