# Supplementary material for: Embracing the Future of Medical Education With Large Language Model–Based Virtual Patients: Scoping Review
Source: J Med Internet Res. 2025 Nov 13;27:e79091. doi: 10.2196/79091 (PMC12661241; doi:10.2196/79091)
Supplement: Multimedia Appendix 2 [file jmir_v27i1e79091_app2.docx]

**Table S1. Search strategy.**

| **Database** | **Query** |
| --- | --- |
| **WoS CC** | ((TS=("artificial intelligence" OR "AI" OR "generative artificial intelligence" OR "generative AI" OR "generative pre-trained transformers" OR "large language model" OR "LLM" OR "natural language processing" OR "ChatGPT" OR "Chatbot*" OR "GPT" OR "Bing" OR "Prometheus" OR "Bard" OR "PaLM" OR "Pathways Language Model" OR "LaMDA" OR "Language Model for Dialogue Applications" OR "Llama" OR "Large Language Model Meta AI")) AND TS=("virtual patients" OR "virtual patient" OR "digital patients" OR "digital patient" OR “avatar*” OR "virtual standardized patients" OR "virtual standardized patient" OR "virtual simulation" OR "virtual simulations" OR "standardized patients" OR "standardized patient" OR "simulated patients" OR "simulated patient" OR "standard patients" OR “standard patient” OR “artificial intelligence patient” OR “virtual patient simulator”)) |
| **Pubmed** | ("virtual patients"[Title/Abstract] OR "virtual patient"[Title/Abstract] OR "digital patients"[Title/Abstract] OR "digital patient"[Title/Abstract] OR "avatar*"[Title/Abstract] OR "virtual standardized patients"[Title/Abstract] OR "virtual standardized patient"[Title/Abstract] OR "virtual simulation"[Title/Abstract] OR "virtual simulations"[Title/Abstract] OR "standardized patients"[Title/Abstract] OR "standardized patient"[Title/Abstract] OR "simulated patients"[Title/Abstract] OR "simulated patient"[Title/Abstract] OR "standard patients"[Title/Abstract] OR "standard patient"[Title/Abstract] OR "artificial intelligence patient"[Title/Abstract] OR "virtual patient simulator"[Title/Abstract]) AND ("artificial intelligence"[Title/Abstract] OR "AI"[Title/Abstract] OR "generative artificial intelligence"[Title/Abstract] OR "generative AI"[Title/Abstract] OR "generative pre-trained transformers"[Title/Abstract] OR "large language model"[Title/Abstract] OR "LLM"[Title/Abstract] OR "natural language processing"[Title/Abstract] OR "ChatGPT"[Title/Abstract] OR "Chatbot*"[Title/Abstract] OR "GPT"[Title/Abstract] OR "Bing"[Title/Abstract] OR "Prometheus"[Title/Abstract] OR "Bard"[Title/Abstract] OR "PaLM"[Title/Abstract] OR "Pathways Language Model"[Title/Abstract] OR "LaMDA"[Title/Abstract] OR "Language Model for Dialogue Applications"[Title/Abstract] OR "Llama"[Title/Abstract] OR "Large Language Model Meta AI"[Title/Abstract]) |
| **Scopus** | ( TITLE-ABS-KEY ( "artificial intelligence" OR "AI" OR "generative artificial intelligence" OR "generative AI" OR "generative pre-trained transformers" OR "large language model" OR "LLM" OR "natural language processing" OR "ChatGPT" OR "Chatbot*" OR "GPT" OR "Bing" OR "Prometheus" OR "Bard" OR "PaLM" OR "Pathways Language Model" OR "LaMDA" OR "Language Model for Dialogue Applications" OR "Llama" OR "Large Language Model Meta AI" ) AND TITLE-ABS-KEY ( "virtual patients" OR "virtual patient" OR "digital patients" OR "digital patient" OR "avatar*" OR "virtual standardized patients" OR "virtual standardized patient" OR "virtual simulation" OR "virtual simulations" OR "standardized patients" OR "standardized patient" OR "simulated patients" OR "simulated patient" OR "standard patients" OR "standard patient" OR "artificial intelligence patient" OR "virtual patient simulator" ) |
| **Embase** | ('artificial intelligence':ti,ab,kw OR 'ai':ti,ab,kw OR 'generative artificial intelligence':ti,ab,kw OR 'generative ai':ti,ab,kw OR 'generative pre-trained transformers':ti,ab,kw OR 'large language model':ti,ab,kw OR 'llm':ti,ab,kw OR 'natural language processing':ti,ab,kw OR 'chatgpt':ti,ab,kw OR 'chatbot*':ti,ab,kw OR 'gpt':ti,ab,kw OR 'bing':ti,ab,kw OR 'prometheus':ti,ab,kw OR 'bard':ti,ab,kw OR 'palm':ti,ab,kw OR 'pathways language model':ti,ab,kw OR 'lamda':ti,ab,kw OR 'language model for dialogue applications':ti,ab,kw OR 'llama':ti,ab,kw OR 'large language model meta ai':ti,ab,kw) AND ('virtual patients':ti,ab,kw OR 'virtual patient':ti,ab,kw OR 'digital patients':ti,ab,kw OR 'digital patient':ti,ab,kw OR 'avatar*':ti,ab,kw OR 'virtual standardized patients':ti,ab,kw OR 'virtual standardized patient':ti,ab,kw OR 'virtual simulation':ti,ab,kw OR 'virtual simulations':ti,ab,kw OR 'standardized patients':ti,ab,kw OR 'standardized patient':ti,ab,kw OR 'simulated patients':ti,ab,kw OR 'simulated patient':ti,ab,kw OR 'standard patients':ti,ab,kw OR 'standard patient':ti,ab,kw OR 'artificial intelligence patient':ti,ab,kw OR 'virtual patient simulator':ti,ab,kw) |
| **IEEEXplore** | ("Index Terms":"virtual patients" OR "Index Terms":"virtual patient" OR "Index Terms":"digital patients" OR "Index Terms":"digital patient" OR "Index Terms":“avatar*” OR "Index Terms":"virtual standardized patients" OR "Index Terms":"virtual standardized patient" OR "Index Terms":"virtual simulation" OR "Index Terms":"virtual simulations" OR "Index Terms":"standardized patients" OR "Index Terms":"standardized patient" OR "Index Terms":"simulated patients" OR "Index Terms":"simulated patient" OR "Index Terms":"standard patients" OR "Index Terms":“standard patient” OR "Index Terms":“artificial intelligence patient” OR "Index Terms":“virtual patient simulator”) AND ("Index Terms":"artificial intelligence" OR "Index Terms":"AI" OR "Index Terms":"generative artificial intelligence" OR "Index Terms":"generative AI" OR "Index Terms":“generative pre-trained transformers” OR "Index Terms":"large language model" OR "Index Terms":"LLM" OR "Index Terms":"natural language processing" OR "Index Terms":"ChatGPT" OR "Index Terms":"Chatbot*" OR "Index Terms":"GPT" OR "Index Terms":"Bing" OR "Index Terms":"Prometheus" OR "Index Terms":"Bard" OR "Index Terms":"PaLM" OR "Index Terms":"Pathways Language Model" OR "Index Terms":"LaMDA" OR "Index Terms":"Language Model for Dialogue Applications" OR "Index Terms":"Llama" OR "Index Terms":"Large Language Model Meta AI") |

**Table S2. Data extraction.**

| **Study Characteristics** |  |
| --- | --- |
| ID | Unique ID assigned to each study. |
| Author | The first author of the study. |
| Year | The year in which the study was published. |
| Country of publication | The country of the first author’institution. |
| Study aim | The aim of each study. |
| Study design | Type of study design. |
| Key conclusion | The key conclusions drawn from each study. |
| Key limitation | The key limitations of each study. |
| Bias | The apparent biases present in the design of each study. |
| **Data of design** |  |
| Large language models | The models used in each study (e.g., GPT-4, GPT-4o). |
| Prompts | The instructions designed to achieve a more realistic scenario and meet the intended objectives. |
| Fine-tuning | Continuing the training of a model using domain-specific or task-specific data to better adapt it to a particular application scenario. |
| Technology and tools | The software and hardware devices integrated to simulate the patient in the model. |
| Participant | The specific characteristics of the experimental subjects in the study. |
| Sample Size | Number of participantsused. |
| Avatar | The representation of the model. |
| Simulated Patients | The specific patient types simulated by the model. |
| Medical specialty | The primary disciplinary scope involved in each study. |
| Medical context and tasks | The scenarios of the research design and the specific tasks performed (e.g., communication training in an outdoor emergency situation). |
| **Data of evalation** |  |
| Evaluation domain and tools | An overview of the tools used to assess the LLM-VPs in the study (e.g., scales, questionnaires, etc.), and the specific aspects evaluated (such as user experience, learning outcomes). |
| Evaluators | The entities responsible for assessing user experience and learning outcomes (e.g., experts). |
| **Quality assessment** | |
| MERSQI | A validated tool for evaluating the quality of quantitative medical education research, has a scoring range from 5 (indicating the lowest quality) to 18 (indicating the highest quality). |
| QualSyst standard | A tool for evaluating the qualitative research, includes a checklist of 10 criteria for assessing qualitative studies. The score, representing the ratio of obtained points to the maximum possible score, ranges from 0 (indicating the lowest quality) to 1 (indicating the highest quality). |
