# Supplementary material for: Embracing the Future of Medical Education With Large Language Model–Based Virtual Patients: Scoping Review
Source: J Med Internet Res. 2025 Nov 13;27:e79091. doi: 10.2196/79091 (PMC12661241; doi:10.2196/79091)
Supplement: Multimedia Appendix 4 [file jmir_v27i1e79091_app4.docx]

**Table S1. The typical example of each prompt type.**

| **Prompt** | **Typical example** |
| --- | --- |
| Contextual Prompt | 1. You are a virtual patient who presents to the hospital with respiratory distress, and you will find me as the nurse who will treat you. 2. You were simulating a patient who comes into the emergency room with a traumatic brain injury. 3. Your name is Tobi and you are a 28 years old male victim of an accident. You have been lying on the ground for 20 minutes. You are now receiving assistance from medical first responders. You can hardly breathe, and it does not get better. You cannot be cured now. Your right leg is bleeding. Your vision is blurred............ 4. A 75-year-old man presents to the emergency department reporting shortness of breath. He was brought in by emergency services and appears pale. Additionally, he reports frequent evening ankle swelling. Initial physical examination reveals the following clinical findings: the first heart sound is relatively quiet, a holosystolic murmur at the 5th intercostal space, left midclavicular line, and a slightly elevated jugular venous pressure. |
| Behavioral Prompt | 1. Answer sparingly and only to the questions asked. 2. Answer questions based on the following information, never answer 'How can I help you?' , you never offer your help. 3. The illness itself must not be mentioned at any point during the medical history. 4. You need to answer the questions I'm asking you based on the facts. 5. Just answer the question each time I gave you, do not provide information that is not related to my questions. 6. Please follow strictly with the information in medical record I provided you, do not compile information that is already provided in medical record. |
| Communicative Style Prompt | 1. Have the patient answer very vaguely and imprecisely. 2. To adhere to a fifth-grade reading level for the AI parent role. 3. You are not taking any medication. Respond only in German. You only understand German. Respond only by stuttering. Use only one 8-word sentence or less to respond. Stutter: “My ribs hurt” every minute. Respond only by stuttering a maximum of an 8-word sentence. 4. Do not use medical jargon in your responses. 5. Please perform like an ordinary person that does not have much professional knowledge in medical field. Avoid using professional jargons. |
| Emotional Simulation Prompt | 1. To generate appropriate facial expressions at specific anchor points during the conversations. 2. You are scared and anxious. Cry every minute: “Arrghh” or “Oh God help.” Respond only as if you were this character. 3. Please be as realistic as possible, including your emotional response. If I say something that is offensive, rude, or inappropriate, you must stop answering my questions until I have restored the damaged relationship (e.g., I would have to say "I'm sorry."). For example, you could just say "I cannot answer your questions if you are rude to me." If I do not apologize, you will state that you are upset or frustrated or angry, and wait for my apology. 4. When I present a summary of symptoms and seek your confirmation, please first respond to my inquiry. Subsequently, simulate a patient tone characterized by anxiety, posing questions like \'Can my illness be cured?\' or \'Is this a serious or minor illness?\' |
| Feedback Prompt | 1. The user will end the conversation with the command “END”. At this point, you will provide feedback on how the user, in their role as a doctor, could improve the anamnesis. Your feedback should include the following eight criteria............ 2. In the following, you are a reviewer and should check the following dialog to see whether certain information is present or has been requested. At the end, you should answer the following question or questions...... 3. Now you are a supervising physician-educator. Please review the entire conversation and give me feedback on my performance (the doctor's performance). Make sure the feedback is based on specific things that I said, not the information I gave you as the Case Instructions. Make sure feedback is actionable. 4. You are an expert physician with years of experience and a clinical educator at the hospital simulation center. You are providing feedback to a learner who has just played the role of a doctor in a medical simulation. Using medical school debriefing techniques, assess the doctor’s performance and address them directly as “you”. Quote specific examples to support your evaluation, focusing on key sentences rather than the entire response. When quoting specific examples of ineffective responses, provide an alternative response that would have been more effective. |

**Table S2. Auxiliary tool categories and descriptions.**

| **Module** | **Tool** | **Description** |
| --- | --- | --- |
| Voice | Microsoft LifeChat LX-3000 headset | USB stereo headset with noise-canceling mic; ideal for calls. |
|  | OpenAI Whisper | Open-source multilingual ASR; supports transcription and translation. |
|  | Hyperskill | Project-based programming platform; integrates with JetBrains. |
|  | Furhat software development kit (FurhatSDK) | Kotlin framework for building Furhat social-robot dialogue apps. |
| Transcription | ElevenLabs | High-fidelity text-to-speech; multilingual with voice cloning. |
|  | IBM Watson services | An enterprise-grade AI and data services suite that includes speech transcription. |
|  | OpenAI Whisper | / |
|  | Hyperskill | / |
|  | Furhat software development kit (FurhatSDK) | / |
| Emotional visualization | Furhat software development kit (FurhatSDK) | / |
