# Supplementary material for: Embracing the Future of Medical Education With Large Language Model–Based Virtual Patients: Scoping Review
Source: J Med Internet Res. 2025 Nov 13;27:e79091. doi: 10.2196/79091 (PMC12661241; doi:10.2196/79091)
Supplement: Multimedia Appendix 6 [file jmir_v27i1e79091_app6.docx]

**Summary of LLM-VPs Applications in Medical Training – Tasks, Integration, and Effectiveness**

| **Reference** | **Summary of task** | **Performance/result** | **Research design** | **Number of participants** | **Participant demographics** |
| --- | --- | --- | --- | --- | --- |
| [42] | To valuate the effectiveness of ChatGPT as a virtual patient in nursing simulation training, with a focus on students' user experience and assessing students' virtual patient interaction skills. | Students gave ChatGPT high ratings for accessibility and engagement, finding it valuable for training. Their interaction skills were positively linked to overall performance, with particular emphasis on the clarity, relevance, and usefulness of responses. | Observational Study | 12 | Twelve second-year master's students participated, with 70% female and 30% male. The average age was 24 years. All participants had prior medical simulation experience and were using ChatGPT in medical simulation for the first time. |
| [43] | This study was to explore the potential added value of a social robotic VP platform, enriched with an LLM, compared with a conventional computer-based platform for medical students practicing CR skills | students preferred the LLM-powered social robotic platform over the computer-based one, as it offered a more authentic and engaging learning experience for developing clinical reasoning and communication skills. | Observational Study | 23 | Fourteen of 23 students (61%) were male and nine (39%) were female. Seventeen (74%) responded that they had no previous experience with VPs. The students’ mean age was 23.5 (standard deviation: 6.0) years. Twelve of the students (52%) started with the social robotic platform, whereas eleven (48%) started with the computer-based VPs. |
| [20] | This study explored the potential of using Large Language Models (LLM) to simulate patient-doctor interactions and provide structured feedback. | the feedback group, which received LLM-generated feedback, significantly outperformed the control group in clinical decision-making (CDM). The feedback group showed notable improvements in the subdomains of "creating context" and "securing information," while there was no significant improvement in "focusing questions." | randomized controlled trial | 21 | A total of 29 students initially participated in the study. Before randomization, 5 students withdrew, leaving 24 students who underwent randomization. Ultimately, 21 students were included in the analysis, with 3 excluded due to technical issues. The control group consisted of 11 students, and the feedback group had 10 students. The 21 students were aged between 19 and 35, and none had clinical rotation experience. The majority were third-year students. |
| [44] | This study explored the effectiveness of generative AI (ChatGPT) in developing realistic virtual standardized patient dialogues to teach prenatal counseling skills. | 35 role-play scenarios generated 176 unique parent responses, including 268 sentences. 65% of the responses expressed emotions, and about half included multiple sentences or questions. More than half described feelings such as fear or concern. Parent role-plays with low trust in the healthcare system generated more unique sentences. Most responses were realistic, relevant, and could be used in the virtual patient program with minimal modifications. | Observational Study | \ | \ |
| [45] | To evaluate the effectiveness of a Generative Pretrained Transformer (GPT) 4 model to provide structured feedback on medical students’ performance in history taking with a simulated patient. | We analyzed 1,894 question-answer pairs from 106 conversations. In over 99% of cases, GPT-4's responses were medically reasonable. The inter-rater reliability with human evaluators showed "almost perfect" agreement (Cohen κ = 0.832). Lower consistency (κ < 0.6) was found in 8 of 45 feedback categories, where the model's evaluations were too specific or deviated from human judgment. | Observational Study | 106 | A total of 111 students participated, with 5 dropping out due to technical issues, leaving 106 students in the final sample. Among the participants, 78 (73.6%) were female, 25 (23.6%) were male, and 3 (2.8%) identified as non-binary. The average age of participants was 22.8 years (SD = 3.7). 93% of participants (N = 99) were in their third year of medical school, while the remaining participants were in their first year (2/106, 2%), second year (1/106, 1%), or fourth year (3/106, 3%). One student provided an unreasonable answer (1/106, 1%). |
| [21] | To evaluate the feasibility and effectiveness of GPT-based large language model (LLM) driven chatbots as simulated patients for history-taking training in medical education. | In the study, 826 question-answer pairs were recorded, with most focusing on history-taking. The majority of responses (94%) were based on the provided script. For unscripted questions, over 50% of answers included fictitious information. Nearly 98% of answers were deemed plausible, with only 2% being implausible due to issues like socially desirable answers and minor errors. Overall, participants reported a positive user experience, with a high satisfaction score. | Observational Study | 28 | The study involved 28 students, 24 of whom were female and 4 were male. Their ages ranged from 19 to 31 years (mean 23.4 years, standard deviation 2.9 years). Twenty-six students (92.9%) were studying clinical medicine, while 2 (7.1%) were studying midwifery. The semester ranged from the second to the tenth, with one participant (3.6%) in their final year. |
| [46] | To study how the integration of GPT-based AI in a mixed reality (MR)–VP could support communication training of  medical first responders(MFRs). | The usability assessment of the VP showed generally positive ratings, especially in habitability and likability. MFRs found it natural to determine the VP's physiological state through verbal communication, but noted that the VP could not initiate the conversation. | Observational Study | 24 | performed the study with 24 MFRs (women: n=4, 17% and men: n=20, 83%) aged 19 to 50 (mean 30.61, SD 9.21) years. |
| [47] | This study evaluated the possibility of using a ‘no-code’ generative AI solution to create 2D and 3D virtual avatars | The tool performed well in terms of intuitiveness and user-friendliness, with most users finding it easy to use and intuitive. The accuracy in simulating patient responses and behavior was also recognized, with the majority of users considering it to be highly accurate. A large proportion of respondents (around 87%) reported feeling comfortable and confident while using the tool. | Observational Study | 15 | There were 15 participants, including 11 consultants and 4 junior doctors |
| [48] | To investigate whether medical students’ interview skills could be improved by engaging with AI-simulated patients using large language models, including the provision of feedback. | The study found that using AI-simulated patients improved medical students' interview skills, leading to higher scores in medical interview evaluations, though it is best used as a supplement to traditional methods due to limitations in nonverbal communication training. | Nonrandomized Controlled Trial | 145 | The AI group, which received LLM-based simulation education, consisted of 35 students from the 87 who agreed to participate in the study, while the control group had 110 students. The AI group included 20 males and 15 females, with an average age of 22 and an average GPA of 2.9. The control group consisted of 34 females and 76 males, with an average age of 23 and an average GPA of 2.7. |
| [49] | To examine whether ChatGPT can be used to train empathic history taking while fostering students’ subjective autonomy. | The study found that ChatGPT can be used to practice empathic history taking, with students reporting high autonomy. While empathic interactions were limited, ChatGPT proved useful for conducting comprehensive history taking, suggesting its potential as a supplement to traditional training. | Observational Study | 35 | Thirty-five students participated in the study, but due to technical issues, the chat records of 7 students were lost. As a result, 28 chat protocols were considered valid. |
| [50] | to explore the potential added value of a social robotic VP platform combined with an LLM compared to a conventional computer-based VP modality for clinical reasoning (CR) training of medical students. | The social robotic platform was seen as more authentic and provided a better overall learning experience compared to the computer-based platform. Students found the robot superior for training communication, emotional, and crisis response skills. However, limitations in technical and user aspects were noted, with suggestions for improvements such as better facial expressions and more varied virtual patient scenarios. | Observational Study | 15 | Among the 15 students, 6 were female and 9 were male. A total of 11 students (73%) reported no prior experience with the VP platform. The average age of the students was 23.9 years (SD = 4.8 years). |
| [51] | To explore whether VP driven by large language models (LLMs) can generate authentic conversations, accurately reflect patient preferences, and provide personalized feedback on clinical presentations. | The conversation cost of GPT-4.0-Turbo is higher than that of GPT-3.5-Turbo, but its conversation ratings are excellent, with high scores for authenticity, user experience, and feedback quality, and minimal score fluctuations. When using GPT-4.0-Turbo, the alignment between the doctor's preferences and expectations is higher, indicating it better meets expectations. Compared to GPT-3.5-Turbo, GPT-4.0-Turbo offers superior conversation quality, with human-generated dialogues also receiving higher ratings. No bias was observed in any conversation, demonstrating fairness and objectivity. | Observational Study | 3 | A board-certified internal medicine physician, 2 investigators |
| [52] | The study investigates whether a chatbot-based training program can influence the types of questions asked by participants. | The study showed that using an AI-driven child chatbot avatar for training did not significantly improve healthcare professionals' interview techniques. Despite feedback, there was no notable increase in the use of open-ended questions. Direct feedback had minimal impact, and participants rated the chatbot as less logical. The study highlighted the need for more realistic, context-rich training scenarios and immediate feedback to improve effectiveness. | Observational Study | 22 | A total of 24 individuals were recruited, with 2 excluded due to lack of consent or incomplete participation. Ultimately, 22 participants were included, consisting of 5 doctors, 6 nurses, 9 dentists, and 2 dental hygienists. There were 5 males and 17 females, with ages ranging from 30 to 59 years. |
| [53] | The study aimed to assess intern physicians' competencies in clinical case management, including problem-solving, clinical reasoning, and crisis management, using ChatGPT-4.0 as a virtual standardized patient. It also explored ChatGPT-4.0's potential as a tool for evaluating these skills in medical training. | Participants were satisfied with the AI-powered standardized patient process and were open to continuing similar practices. However, disconnection issues and language processing challenges sometimes affected the application process. | Observational Study | 21 | The study involved 21 participants (8 female, 13 male) with an average age of 24 ± 1.03 years (range 23–26). |
| [54] | The study investigates the effectiveness of ChatGPT-based virtual patients for student interview training and evaluates students' user experience. | The evaluation revealed that students preferred the AI-driven method for its immersive and interactive nature. Over 80% rated the chatbot's language ability and professional accuracy positively, with most students perceiving it as closely resembling a real anamnesis interview. Additionally, students favored this AI-based training over traditional in-person role-plays. | Observational Study | 28 | There were 28 participants, 24 females and 4 males, aged between 18 and 55. More than half of the students were studying nursing, and 86% of the participants had prior experience with anamnesis collection. |
| [55] | The study aims to explore ChatGPT’s viability and performance as a standardized patient, using prompt engineering to refine its accuracy and use in medical assessments. | The feasibility test confirmed ChatGPT's ability to simulate a standardized patient, accurately distinguishing between poor, medium, and good medical inquiries. The revised prompt improved realism, clinical accuracy, and adaptability, reducing scoring discrepancies. ChatGPT's score accuracy increased by 4.926 times compared to unrevised prompts. Performance differences between test groups using different language combinations were negligible. | Observational Study | \ | \ |
| [56] | Exploring the effect of AI chatbots in improving dental education through medical history collection, with a focus on the chatbot's performance in communication during the history-taking process. | In the observation of clinical educators playing the role of patients, only 2 out of 13 students actively asked questions, while the engagement of students interacting with the chatbot was 100%. Most students believed the chatbot helped improve their skills and provided more practice opportunities, though they remained cautious about its accuracy. It was well-received by both staff and advanced students. | Observational Study | 13 | 13 third-year Doctor of Dental Medicine students |
| [18] | Investigating the performance of ChatGPT as an interactive virtual patient. | The patient-clinician conversations using GPT-4.0 to be authentic, engaging, and appropriately unpredictable. | Not clinically validated | \ | \ |
| [33] | To explore the quality of responses provided by virtual patient programs using generative artificial intelligence (AI) technology during the medical history collection process and assess their feasibility. | The chatbot generated a series of question-answer pairs, with a small percentage of responses deemed unclear, inaccurate, or missing important information. Participants generally found the AI's answers relevant, effective, and accurate, though they felt the fluency could be improved. Using generative AI to record virtual patient histories is feasible, but the responses need to be clearer and more natural. | Observational Study | 5 | Three AI experts and two medical educators |
| [40] | This study aim to set the stage for a comprehensive evaluation of ChatGPT version 3.5 utility as a virtual patient for MS training, diving into its current benefits plus limitations and offering some solutions for the latter. | ChatGPT-3.5 is a useful tool for medical education, providing a platform for clinical practice. However, it cannot replace real patient interactions due to the lack of physical exams and non-verbal cues. Effective use requires careful prompt creation and clinician guidance. | Not clinically validated | \ | \ |
| [32] | This study aims to describe our developmental process and lessons learned for creating a GPT-4–driven VSP, and  assess GPT-4’s ability to generate appropriate VSP responses to learners during spoken conversations and provide appropriate feedback on learner performance. | In-depth interviews help determine the appropriate timing, methods, and communication protocols between primary care physicians and patients during breast cancer screening. The virtual patient asks reasonable questions about mammogram results and responds to the learner with an appropriate emotional tone. The feedback generated by GPT-4 successfully identifies the learner's strengths and areas for improvement, but occasionally misjudges their adherence to communication protocols. | Observational Study | \ | \ |
| [39] | To explore the types of questions interviewers asked of the child avatar, how the avatar responded to the questions, and how interviewers perceived and experienced the interview and avatar | The virtual character appears somewhat realistic in terms of voice, appearance, and expression, and participants felt relatively comfortable using the system. Interaction with the virtual character may reflect real-world investigative interview practices. Participants considered the virtual character a valuable tool for enhancing forensic interview skills. | Observational Study | 12 | Medical student, resident, physician and nurse practitioner. |
| [41] | To verify the accuracy of the information generated by ChatGPT simulating SP. | Out of 10 cases, 2 were rated as 10 points and 6 were rated as 9 points, the remaining 2 cases were rated as 8 points. | Observational Study | / | / |
